# Lipid metabolic plasticity in glioblastoma: mechanisms, tumor microenvironment remodeling, and therapeutic opportunities

**DOI:** 10.3389/fonc.2026.1888793

**Published:** 2026-07-21

**Authors:** Weiqi Wu, Minying Liu, Qi Lv, Zixun Ming, Longchang Song, Junsheng Chu

**Affiliations:** 1 Department of Neurosurgery, Beijing Tiantan Hospital, Capital Medical University, Beijing, China; 2 Department of Pain Management, Beijing Tiantan Hospital, Capital Medical University, Beijing, China

**Keywords:** glioblastoma, lipid metabolism reprogramming, metabolic plasticity, therapeutic resistance, tumor microenvironment

## Abstract

Glioblastoma (GBM) is the most aggressive primary brain tumor and remains associated with poor prognosis despite multimodal treatment. Increasing evidence indicates that lipid metabolic reprogramming is a critical hallmark of GBM progression and therapeutic resistance. Beyond supporting membrane biosynthesis and energy production, dysregulated lipid metabolism profoundly influences tumor plasticity, oxidative stress adaptation, immune suppression, stemness maintenance, and ferroptosis sensitivity within the tumor microenvironment. GBM cells dynamically rewire lipid metabolic pathways through enhanced *de novo* lipogenesis, fatty acid uptake, cholesterol remodeling, lipid droplet accumulation, and fatty acid oxidation to adapt to hypoxia, nutrient deprivation, and therapeutic stress. In parallel, lipid metabolic interactions between tumor cells and immune components, including tumor-associated macrophages and exhausted T cells, further contribute to immunosuppressive microenvironment formation and resistance to chemotherapy, radiotherapy, and immunotherapy. Recent advances in spatial transcriptomics, single-cell metabolomics, and multi-omics integration have substantially improved understanding of lipid metabolic heterogeneity in GBM. These emerging technologies have also facilitated the identification of novel metabolic vulnerabilities and therapeutic targets. In this review, we summarize current insights into lipid metabolic plasticity in GBM and discuss the mechanistic links between lipid metabolism, tumor microenvironment remodeling, ferroptosis regulation, and therapeutic resistance, highlighting potential opportunities for precision metabolic therapy in GBM.

## 1 Introduction

Glioblastoma (GBM) is the most aggressive primary malignant brain tumor in adults and remains associated with extremely poor clinical outcomes despite multimodal treatment strategies (1, 2). Current standard therapy, including maximal safe surgical resection followed by radiotherapy and temozolomide (TMZ) chemotherapy, provides only limited survival benefits, with most patients eventually developing tumor recurrence and therapeutic resistance. The highly invasive nature and pronounced intratumoral heterogeneity of GBM highlight the need to understand adaptive biological programs that enable tumor progression under dynamic microenvironmental stress. Increasing evidence suggests that metabolic reprogramming is a fundamental hallmark of GBM and plays essential roles in tumor proliferation, invasion, immune evasion, and therapeutic adaptation. However, metabolic rewiring in GBM is increasingly recognized as a dynamic and reversible process that determines cellular state transitions rather than static pathway activation. Although glucose metabolism and the Warburg effect have historically received the greatest attention in cancer metabolism research, lipid metabolism has recently emerged as another critical metabolic dependency in GBM. Lipids not only function as structural components of cellular membranes and energy reservoirs, but also participate in intercellular signaling processes that shape tumor–microenvironment interactions in glioblastoma. Under metabolic stress conditions such as hypoxia and nutrient deprivation, GBM cells exhibit remarkable lipid metabolic plasticity that enables dynamic adaptation to hostile tumor microenvironmental conditions. This adaptive rewiring includes enhanced *de novo* lipogenesis (DNL), increased uptake of exogenous fatty acids and cholesterol, activation of fatty acid oxidation (FAO) pathways, and accumulation of lipid droplets (LDs). Aberrant lipid metabolism contributes not only to rapid tumor growth and membrane biosynthesis, but also to redox homeostasis, stemness maintenance, immune suppression, and resistance to chemotherapy and radiotherapy. Notably, lipid metabolic alterations are not restricted to tumor cells but also occur in tumor-associated immune and stromal compartments, including macrophages, T cells, and glioma stem-like cells, thereby contributing to a lipid-enriched tumor microenvironment that supports GBM progression. Targeting lipid metabolic pathways therefore represents a promising therapeutic strategy that may simultaneously impair tumor growth, overcome therapeutic resistance, reshape the tumor immune microenvironment, and enhance treatment sensitivity. Several recent reviews have summarized lipid metabolism in GBM from the perspective of therapeutic targets, key metabolic enzymes, and candidate inhibitors. In contrast, the present review uses lipid metabolic plasticity as the central organizing concept and focuses on how GBM cells dynamically reallocate lipid fluxes in response to hypoxia, nutrient deprivation, immune pressure, and therapeutic stress. Rather than cataloging lipid metabolic targets alone, we integrate *de novo* lipogenesis, exogenous lipid uptake, cholesterol dependent membrane remodeling, lipid droplet mediated stress buffering, ferroptosis regulation, and immune microenvironment remodeling into a unified adaptive model. This perspective may help clarify how lipid metabolism supports not only tumor growth but also spatial heterogeneity, immune escape, and treatment resistance in GBM.

## 2 Lipid metabolic plasticity in glioma cells

**Figure 1:**
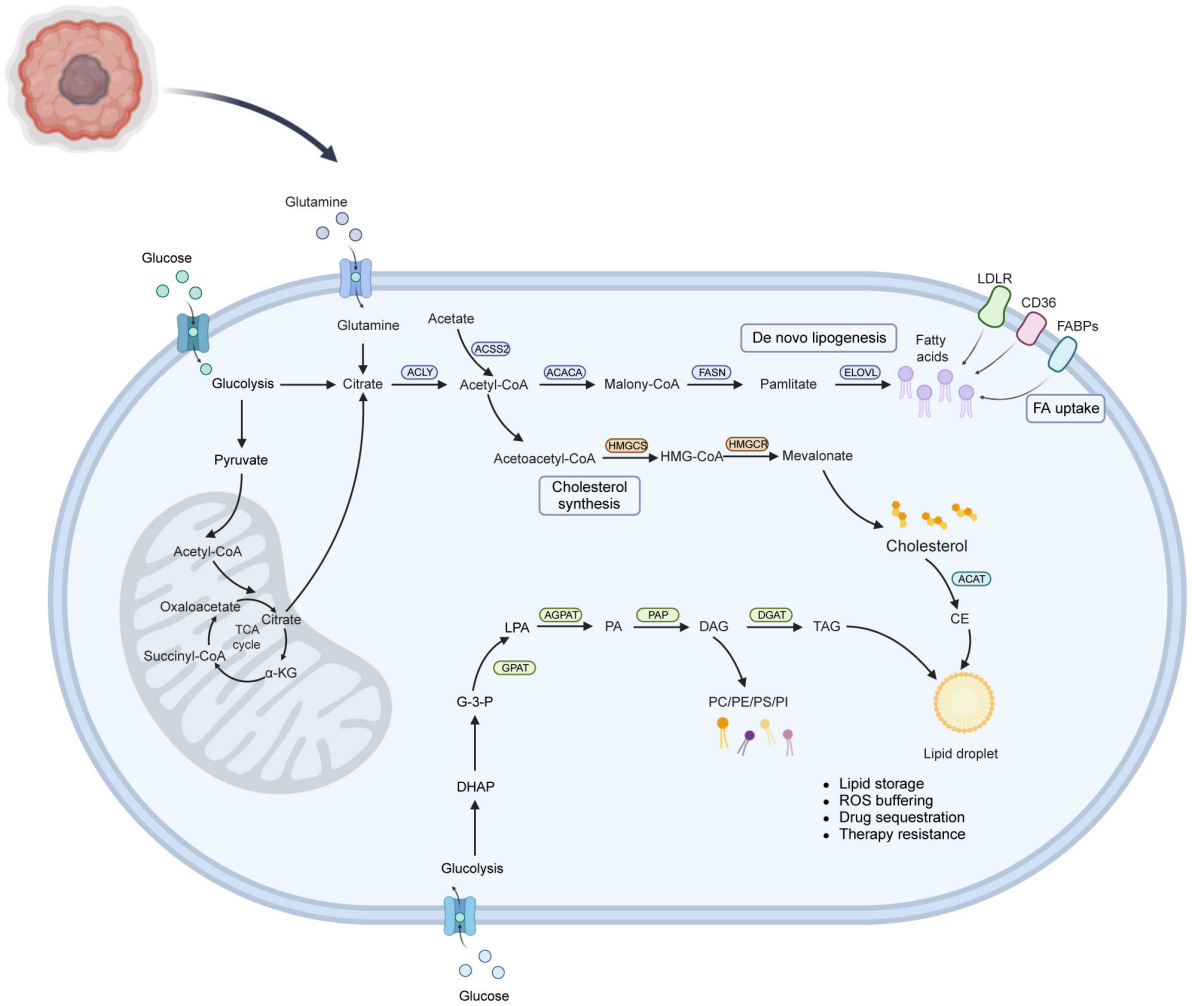
Integrated overview of lipid metabolic pathways in glioblastoma cells. This figure summarizes the major lipid metabolic pathways in GBM cells. Glucose, glutamine, and acetate serve as key carbon sources for lipid biosynthesis through glycolysis, the tricarboxylic acid (TCA) cycle, and acetyl-CoA generation. *De novo* lipogenesis mediated by ACLY, ACACA, and FASN promotes fatty acid synthesis, whereas extracellular lipid uptake is facilitated by LDLR, CD36, and FABPs. The mevalonate pathway further supports cholesterol biosynthesis and esterification. In parallel, phospholipid synthesis and triglyceride formation contribute to membrane remodeling and lipid droplet accumulation. Lipid droplets function as dynamic reservoirs for lipid storage, ROS buffering, drug sequestration, and therapy resistance. Collectively, these metabolic adaptations support tumor growth, metabolic flexibility, and survival under therapeutic stress in GBM.

Lipid metabolism has emerged as a central component of tumor intrinsic metabolic plasticity in glioma, allowing tumor cells to dynamically redistribute lipid fluxes among biosynthesis, uptake, oxidation, storage, and membrane remodeling in response to oxygen fluctuation, nutrient limitation, and therapeutic stress (3). In this review, lipid metabolic plasticity is defined not simply as increased lipid metabolism, but as the capacity of GBM cells to switch between distinct lipid handling programs according to local microenvironmental constraints. This adaptive property is particularly evident during disease progression and treatment exposure, as key lipid metabolic enzymes differ substantially among GBM cell states and recurrent GBM can maintain lipid metabolic activity after TMZ treatment, suggesting that fatty acid metabolic remodeling contributes to therapeutic adaptation (4). Therefore, rather than describing lipid metabolism as a collection of isolated enzymes or therapeutic targets, this section focuses on three interconnected adaptive modules: coordinated *de novo* lipogenesis and exogenous lipid uptake, cholesterol dependent membrane remodeling, and lipid droplet mediated stress buffering. Together, these adaptive modules enable GBM cells to balance membrane production, ATP generation, redox control, ferroptosis sensitivity, and survival signaling under hostile microenvironmental conditions (**Figure 1**) (5).

### 2.1 DNL and lipid uptake

Compared with normal brain tissue, glioma tissues display significantly enhanced lipid synthesis, and nuclear magnetic resonance spectroscopy-based analyses have demonstrated that lipid resonance spectra correlate with GBM malignancy grade. In GBM, *de novo* lipogenesis (DNL) and exogenous lipid uptake should be viewed as complementary lipid acquisition programs rather than independent metabolic pathways. Under nutrient replete conditions, GBM cells can use glucose-derived intermediates, citrate, acetate, and other carbon sources to generate cytosolic acetyl-CoA, which provides the substrate pool required for fatty acid synthesis. Acetyl-CoA is subsequently converted into malonyl-CoA by acetyl-CoA carboxylases (ACCs), thereby supporting fatty acid synthase (FASN) dependent production of saturated fatty acids (6, 7).Within this adaptive network, FASN is not only a lipogenic enzyme but also a stress response node linking membrane biosynthesis, tumor growth, and treatment adaptation. FASN is markedly upregulated in GBM and its expression positively correlates with glioma World Health Organization grade, suggesting that enhanced lipogenesis contributes to malignant progression (8, 9). Radiation exposure can further increase FASN expression, leading to fatty acid and lipid droplet accumulation that suppresses endoplasmic reticulum stress and inhibits apoptosis (10). Therefore, rather than indicating only a druggable enzyme, FASN dependent lipogenesis may reflect a broader adaptive program that helps GBM cells preserve membrane homeostasis and survival signaling during therapeutic stress. Besides endogenous lipid synthesis, GBM cells can actively acquire exogenous lipids from the tumor microenvironment. This scavenging program becomes particularly relevant under hypoxia, nutrient limitation, blood-brain barrier disruption, and therapeutic pressure, when endogenous lipid synthesis alone may be insufficient to maintain lipid demand. GBM cells enhance extracellular fatty acid uptake through lipid transport and trafficking proteins such as CD36, fatty acid transport proteins (FATPs), and fatty acid-binding proteins (FABPs) (11, 12). Stable isotope tracing experiments further demonstrated enrichment of exogenous fatty acids in glioma tissues compared with normal brain tissues, supporting the concept that glioma cells use environmental lipids as metabolic substrates (13). This phenomenon may partly result from disruption of the blood-brain barrier, which increases lipid accessibility within the glioma microenvironment. After uptake, fatty acids are converted into acyl-CoA by long-chain acyl-CoA synthetases (ACSLs), allowing their mitochondrial transport through carnitine palmitoyltransferase 1 and subsequent utilization through fatty acid oxidation (FAO) (14). This process provides an alternative bioenergetic route when glycolytic metabolism is constrained. GBM cells initially rely heavily on glycolysis, but inhibition of glycolytic metabolism can induce a compensatory shift toward oxidative phosphorylation driven by elevated FAO (15). Acyl-CoA binding protein further facilitates FAO by transporting medium- and long-chain acyl-CoAs toward mitochondria, thereby supporting tumor proliferation and invasion. In addition to its bioenergetic function, FAO may contribute to immune evasion, as FAO inhibition downregulates CD47 transcription and impairs tumor growth and immune escape in GBM models (16). Among lipid trafficking proteins, FABP7 represents a state dependent mediator of lipid handling in GBM rather than only a candidate therapeutic target. FABP7 participates in fatty acid uptake, intracellular transport, storage, and metabolism and is preferentially expressed in specific GBM cellular states (11). Inhibition of FABP7 reduces lipid uptake and lipid droplet formation, sensitizes GBM cells to glucose deprivation, and suppresses migratory capacity (17). FABP7 also contributes to hypoxic adaptation, as knockdown of FABP3 or FABP7 reduces lipid droplet formation during hypoxia and impairs tumor growth after hypoxia reoxygenation stress (18). These findings suggest that FABP7 links exogenous lipid acquisition to stress buffering, migration, and metabolic adaptation. Collectively, DNL, exogenous lipid uptake, and FAO form a flexible lipid acquisition and utilization network in GBM. This network allows tumor cells to switch between endogenous synthesis, environmental lipid scavenging, mitochondrial oxidation, and lipid storage according to local nutrient availability, oxygen tension, and treatment exposure. Importantly, recurrent TMZ-resistant GBM cells exhibit increased cholesterol and fatty acid levels and display greater sensitivity to lipid synthesis inhibitors, further supporting the concept that lipid metabolic remodeling contributes to therapeutic adaptation (19).

### 2.2 Cholesterol and membrane remodeling

Cholesterol is a major structural lipid in the central nervous system, where it maintains membrane fluidity, membrane permeability, signal transduction, and myelin stability. Because cholesterol does not efficiently cross the blood-brain barrier, brain cholesterol homeostasis largely depends on local synthesis and tightly regulated intercellular transport (20, 21). In GBM, however, cholesterol metabolism is rewired from a homeostatic neural program into an adaptive membrane remodeling program that supports oncogenic signaling, immune regulation, and therapeutic resistance. A distinctive feature of GBM cholesterol metabolism is the shift from predominant endogenous synthesis toward increased dependence on extracellular cholesterol uptake. GBM cells can suppress *de novo* cholesterol biosynthesis and preferentially acquire cholesterol from the extracellular environment, thereby avoiding intracellular cholesterol overload and limiting activation of ATP-binding cassette transporter A1 (ABCA1), a key cholesterol efflux transporter (22, 23). Low-density lipoprotein receptor (LDLR)-mediated cholesterol uptake represents one major route of this metabolic switch. Importantly, LDLR expression is regulated by EGFR/PI3K/Akt signaling, indicating that oncogenic signaling and cholesterol acquisition are functionally coupled in GBM. Higher LDLR expression has also been associated with increased expression of immune checkpoint molecules such as CTLA4 and LAG3, suggesting that cholesterol uptake may contribute not only to tumor cell survival but also to the formation of an immunosuppressive tumor microenvironment (24). Sterol regulatory element-binding proteins (SREBPs), particularly SREBP1 and SREBP2, coordinate cholesterol uptake, biosynthesis, and lipid homeostasis in GBM (25, 26). Rather than functioning as isolated metabolic regulators, SREBPs integrate nutrient availability, oncogenic signaling, and membrane lipid demand. Cholesterol biosynthetic intermediates may further support tumor progression by activating signaling pathways such as Akt and extracellular signal-regulated kinase (27). These findings suggest that cholesterol metabolism contributes to GBM plasticity not only by supplying membrane lipids but also by reshaping signaling networks that sustain malignant cell states. One of the most important consequences of cholesterol remodeling in GBM is the maintenance of cholesterol-rich lipid rafts within the plasma membrane (28). Lipid rafts are specialized membrane nanodomains that organize oncogenic receptors and membrane-associated signaling complexes. Activation of the PI3K/Akt pathway depends on proper recruitment of Akt to cholesterol-rich membrane domains, indicating that membrane lipid composition can directly regulate oncogenic signaling intensity (29). EGFR and EGFRvIII signaling are also influenced by raft integrity, which may affect receptor docking, spatial clustering, and downstream pathway activation. In GBM, lipid raft organization has been linked to invasive signaling programs, including CD44-associated membrane states. However, evidence linking CD133-positive cancer stem-like cells to lipid raft dependence has mainly been derived from pancreatic cancer models. Therefore, this mechanism should be presented as cross-cancer stemness-related evidence rather than direct GBM evidence, and its relevance to GBM stem-like cells requires further validation (30). Membrane remodeling also intersects with inflammatory signaling. Flotillin-2, an important lipid raft component, contributes to tumor-promoting inflammatory pathways, and its depletion reduces NF-κB expression in GBM models (31). Cholesterol esterification and storage provide another layer of adaptive regulation. GBM tissues contain abundant cholesterol esters stored within lipid droplets, and lipophagy-mediated release of these esters can increase intracellular cholesterol availability (32). ACAT1-mediated cholesterol esterification appears particularly relevant to glioma progression, as ACAT1 inhibition suppresses glioma growth in xenograft models (33). These observations connect cholesterol storage, lipid droplet dynamics, and membrane remodeling into a coordinated adaptive system. Oxysterols, oxidized derivatives of cholesterol, further illustrate the context-dependent effects of cholesterol metabolites in GBM. 27-hydroxycholesterol has been reported to promote GBM cell proliferation and malignant phenotypes, whereas activation of liver X receptor signaling by 24-hydroxycholesterol can induce caspase-3/7 activation and apoptosis in GBM cells (34, 35). Therefore, cholesterol metabolites should not be interpreted as uniformly pro-tumorigenic or anti-tumorigenic. Their biological effects may depend on metabolite species, concentration, receptor context, and cellular state. Collectively, these findings demonstrate that cholesterol metabolism and membrane remodeling are tightly interconnected processes in GBM. By regulating extracellular cholesterol uptake, lipid raft integrity, receptor localization, inflammatory signaling, cholesterol ester storage, and oxysterol signaling, GBM cells use cholesterol metabolism to coordinate oncogenic signaling, stemness-associated states, immune evasion, and therapeutic adaptation.

### 2.3 LDs and hypoxic adaptation

Lipid droplets (LDs) are dynamic intracellular organelles that store neutral lipids and regulate lipid trafficking during cellular stress. In GBM, LDs should not be interpreted merely as passive lipid reservoirs, but as spatially organized stress buffering systems that help tumor cells survive under hypoxia, nutrient deprivation, oxidative stress, and therapeutic pressure. Structurally, LDs contain a neutral lipid core enriched in triacylglycerols (TAGs) and cholesteryl esters surrounded by a phospholipid monolayer and lipid-associated proteins (36). Under fluctuating nutrient conditions, surplus fatty acids and cholesterol can be esterified and stored in LDs, whereas LD-derived lipids can be released through lipolysis or lipophagy to support β-oxidation and ATP production during starvation or energy stress (37, 38). The adaptive role of LDs is particularly relevant in the spatially heterogeneous GBM microenvironment. LD accumulation is enriched in hypoxic regions surrounding necrotic areas and in metabolically stressed tumor niches, suggesting that LD biogenesis reflects local microenvironmental constraints rather than uniform lipid storage across the tumor mass (39, 40). Hypoxia-inducible factor-1α (HIF-1α) promotes LD formation by enhancing fatty acid uptake and lipid remodeling. Importantly, hypoxia-induced LD accumulation in GBM appears to rely predominantly on increased exogenous fatty acid uptake rather than *de novo* lipid synthesis, thereby linking LD formation to the lipid acquisition program discussed above (18). FABP3 and FABP7, together with perilipin-2, are important mediators of hypoxia-associated LD formation in GBM cells. Disruption of FABP3, FABP7, or perilipin-2 reduces ATP production, increases reactive oxygen species (ROS), and suppresses GBM cell survival both *in vitro* and *in vivo* (18). These findings indicate that LDs buffer GBM cells against bioenergetic collapse and oxidative damage during hypoxia and reoxygenation stress. A second function of LDs involves protection from lipotoxicity and membrane stress. Unsaturated fatty acids, particularly oleic acid, can be stored within TAG pools and later released from LDs when extracellular lipid availability or oxygen tension becomes limiting (41). Released unsaturated fatty acids can be incorporated into membrane phospholipids to preserve membrane homeostasis, reduce accumulation of toxic saturated lipids, and alleviate endoplasmic reticulum stress (41). DGAT1 and DGAT2 contribute to this adaptive process by catalyzing triglyceride synthesis and promoting LD formation (41). In this context, DGAT-dependent LD biogenesis should be viewed not only as a targetable pathway, but also as a mechanism that permits GBM cells to redistribute fatty acids between storage, membrane maintenance, and oxidative metabolism under stress. LDs also intersect with therapeutic resistance and ferroptosis regulation. Drug-resistant tumor cells frequently exhibit increased LD content, and LDs may sequester lipophilic anticancer agents, thereby reducing intracellular drug bioavailability. Mechanistically, LDs can reduce the availability of oxidizable polyunsaturated fatty acids in membrane phospholipids and may thereby limit lipid peroxidation and ferroptotic damage. Conversely, impaired LD formation or altered lipid partitioning may increase oxidative stress and sensitize GBM cells to ferroptosis-inducing conditions. Thus, LDs connect lipid storage, redox control, ferroptosis sensitivity, and treatment adaptation.

LD biology is also relevant to GBM stem-like states. LDs are enriched in GBM stem-like cells and support survival under nutrient-limited conditions, partly by providing fatty acids for oxidative metabolism during glucose deprivation (17). This suggests that LD-mediated lipid buffering may be especially important in slow cycling, hypoxic, or stem-like tumor cell populations. Collectively, LDs in GBM function as spatially organized metabolic platforms that coordinate stress adaptation, redox homeostasis, ferroptosis balance, therapy resistance, and stemness maintenance. This interpretation also provides a direct rationale for using spatial metabolomics, single-cell metabolic profiling, and multi-omics integration to identify LD-dependent vulnerabilities in specific GBM niches.

## 3 Lipid metabolism shapes the TME

**Figure 2:**
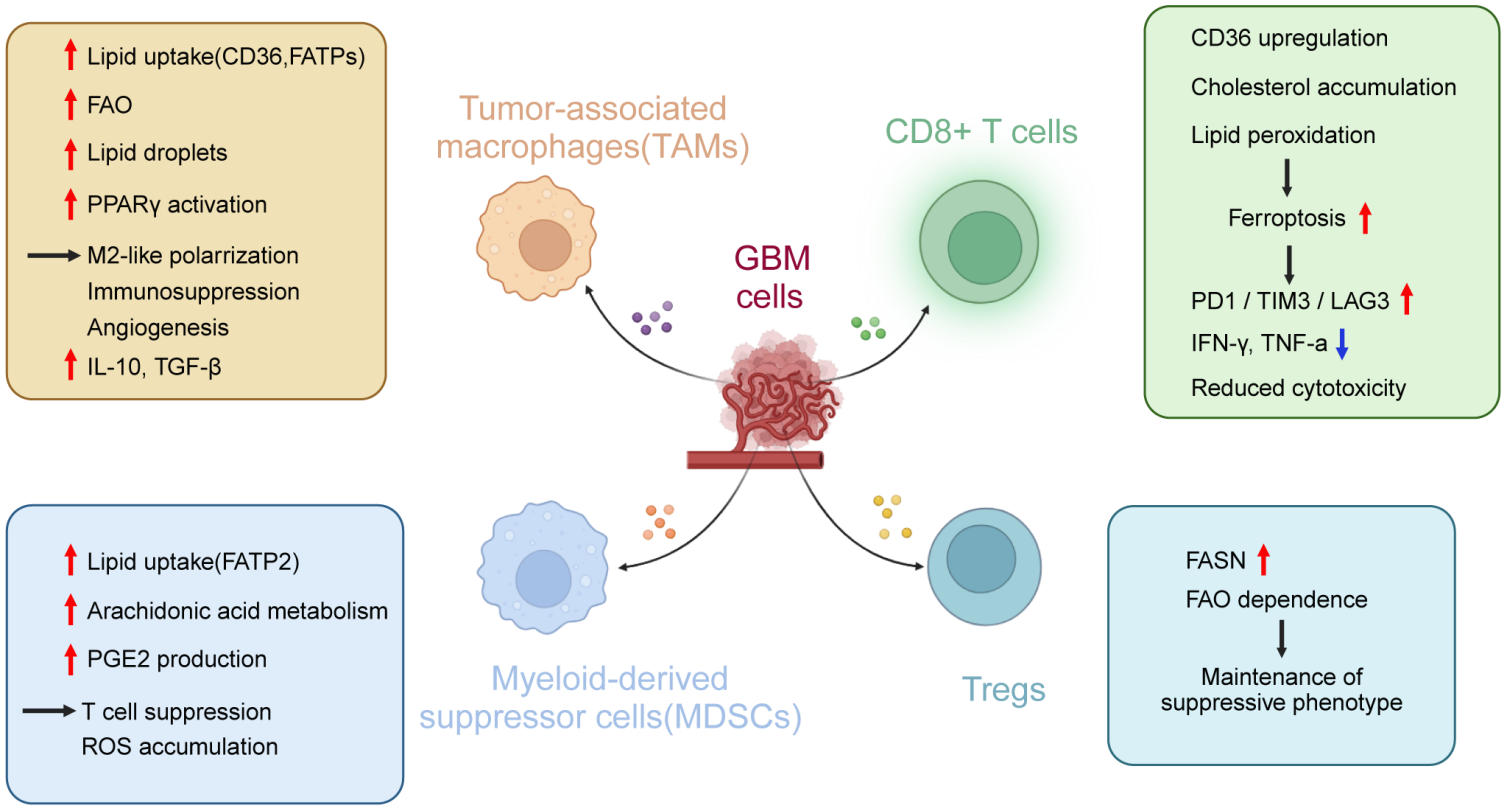
Lipid metabolism shapes the immunosuppressive glioblastoma microenvironment. This figure illustrates how lipid metabolic remodeling contributes to immune suppression within the GBM tumor microenvironment. GBM cells modulate surrounding immune cells through the release of lipid metabolites and signaling molecules. TAMs exhibit enhanced lipid uptake, fatty acid oxidation, lipid droplet accumulation, and PPARγ activation, leading to M2-like polarization and immunosuppressive cytokine production. MDSCs display increased FATP2-mediated lipid metabolism, arachidonic acid metabolism, and ROS accumulation, thereby suppressing T cell activity. CD8+ T cells undergo CD36-mediated lipid uptake, cholesterol accumulation, lipid peroxidation, and ferroptosis, resulting in T-cell exhaustion characterized by increased PD-1, TIM-3, and LAG-3 expression. Regulatory T cells (Tregs) rely on fatty acid synthesis and FAO to maintain their suppressive phenotype. Collectively, lipid metabolic reprogramming promotes immune evasion and tumor progression in GBM.

Glioma-associated macrophages and microglia (GAMs), including resident microglia and infiltrating monocyte-derived macrophages, constitute the predominant immune population within the glioma microenvironment and exhibit remarkable metabolic plasticity (42). Lipid metabolic reprogramming in glioma reshapes the functional phenotype of these cells by regulating fatty acid uptake, cholesterol efflux, lipid droplet accumulation, and phospholipid signaling, ultimately driving immunosuppression, tumor invasion, angiogenesis, and therapeutic resistance (43) (**Figure 2**).

### 3.1 GAMs

GAMs are major cellular components of the GBM microenvironment and include brain-resident microglia and infiltrating monocyte-derived macrophages (44). These myeloid populations are closely associated with tumor progression, immune suppression, angiogenesis, and therapeutic resistance. Rather than representing a fixed M1/M2 binary, GAMs occupy dynamic activation states shaped by tumor evolution, regional hypoxia, acidosis, nutrient deprivation, lipid accumulation, and therapeutic exposure. In this context, lipid metabolic remodeling functions as a tumor-immune adaptation mechanism that links GBM cell intrinsic lipid plasticity to myeloid immune suppression. The GBM microenvironment promotes lipid metabolic adaptations in GAMs, including enhanced fatty acid uptake, fatty acid oxidation (FAO), lipid droplet accumulation, and cholesterol efflux (45, 46). These changes not only provide energy substrates for myeloid cell survival but also regulate transcriptional and inflammatory programs associated with tumor-promoting activity. Immunosuppressive GAM states often show increased reliance on FAO and oxidative phosphorylation, whereas pro-inflammatory myeloid states are more commonly associated with glycolytic metabolism (47). Elevated FAO can enhance mitochondrial oxidative phosphorylation, reactive oxygen species (ROS) production, and downstream STAT6 signaling, thereby supporting M2-like polarization and suppressive activity (48). Peroxisome proliferator-activated receptors (PPARs), especially PPARγ and PPARβ/δ, serve as lipid-sensing transcriptional regulators in this process (49). Activation of PPAR signaling promotes FAO efficiency and induces transcriptional programs associated with M2-like polarization and immune suppression, while polyunsaturated fatty acids such as linoleic acid may act as endogenous PPARβ/δ ligands that further reinforce suppressive myeloid states (50). Lipid uptake is a central mechanism through which the GBM microenvironment educates GAMs. CD36 is one of the major lipid transporters involved in this process and is highly expressed in tumor-associated macrophages (48). Through CD36-mediated uptake, GAMs can internalize extracellular fatty acids, oxidized lipids, and tumor-derived lipid-rich extracellular vesicles (51). This lipid loading promotes the release of immunosuppressive cytokines and chemokines and supports glioma progression. Increased lipid uptake also facilitates lipid droplet accumulation, which provides fatty acids for sustained FAO and oxidative metabolism under nutrient-limited conditions (52). In macrophage and tumor-associated macrophage models, inhibition of lipid droplet formation can shift macrophage polarization from M2-like states toward M1-like states (53). These findings suggest that lipid droplets in GAMs are not passive storage structures but metabolic reservoirs that stabilize suppressive myeloid functions. The FABP family also contributes to lipid trafficking and immune regulation in myeloid cells (54). Different FABP isoforms may exert divergent effects on macrophage function. Evidence from macrophage and tumor-associated macrophage models suggests that epidermal FABP can enhance interferon-β production and recruit immune effector cells, thereby supporting anti-tumor activity (55). By contrast, adipocyte/macrophage FABP can promote tumor progression through activation of the NF-κB/STAT3 signaling axis (56). Although not all of these findings are derived from GBM-specific models, they provide a mechanistic rationale for investigating whether FABP-mediated lipid trafficking determines tumor-promoting or tumor-restraining GAM states in GBM. Phospholipid-derived lipid mediators represent another layer of GAM reprogramming. Arachidonic acid (AA) metabolism generates bioactive mediators such as prostaglandin E2 (PGE2) and leukotrienes, which regulate immune suppression, angiogenesis, and tumor cell migration (57). PGE2 suppresses immune activation and may reinforce an immunosuppressive myeloid niche through multiple signaling pathways. Similarly, 5-lipoxygenase (5-LOX)-dependent leukotriene synthesis in macrophages contributes to tumor-promoting inflammation and immune dysregulation. These lipid mediators provide a direct link between phospholipid remodeling and immune suppression in the GBM microenvironment. Cholesterol handling is also an important immunometabolic checkpoint in GAMs. GBM-associated macrophages frequently exhibit enhanced cholesterol efflux mediated by ATP-binding cassette transporters such as ABCA1 and ABCG1 (58). Cholesterol depletion can promote M2-like polarization and suppress anti-tumor immune signaling, whereas disruption of cholesterol efflux pathways may activate NF-κB signaling and induce a phenotypic shift toward M1-like macrophage states (59). These observations suggest that cholesterol flux, similar to fatty acid uptake and lipid droplet turnover, contributes to the plasticity of GAM phenotypes. Collectively, lipid metabolic remodeling in GAMs should be understood as a reciprocal tumor-immune adaptation program rather than a simple metabolic consequence of macrophage polarization. Fatty acid uptake, FAO, lipid droplet accumulation, FABP-mediated trafficking, phospholipid-derived mediator production, and cholesterol efflux collectively shape suppressive myeloid states in GBM. Therapeutically, these pathways may provide myeloid-directed entry points for reshaping the GBM immune microenvironment, but strategies targeting GAM lipid metabolism should distinguish GBM-specific evidence from cross-cancer myeloid mechanisms and require further validation in GBM-relevant models.

### 3.2 T-cell exhaustion and immunosuppressive metabolism

CD8+ tumor-infiltrating lymphocytes (TILs) are central mediators of anti-tumor immunity, but they progressively acquire an exhausted phenotype during GBM progression (60). T-cell exhaustion is characterized by sustained expression of inhibitory receptors, impaired cytokine production, mitochondrial dysfunction, and reduced cytotoxic activity (61). Within the GBM TME, metabolic stress caused by hypoxia, glucose deprivation, acidosis, and lipid accumulation profoundly reshapes T cell metabolism and function (62). Effector CD8+ T cells normally rely on glycolysis to support proliferation and cytotoxic activity. However, nutrient competition with tumor cells can suppress glycolytic activity in TILs and force a compensatory shift toward lipid utilization and fatty acid oxidation (FAO) (63). This shift may initially help sustain T-cell survival under nutrient-limited conditions, but persistent lipid overload can convert this adaptive response into metabolic dysfunction. Accumulation of oxidized lipids, cholesterol, and long-chain fatty acids promotes reactive oxygen species (ROS) production, lipid peroxidation, mitochondrial stress, and impaired production of anti-tumor cytokines such as interferon-γ (IFN-γ) and tumor necrosis factor-α (TNF-α) (64, 65). In this context, lipid metabolic pressure links nutrient deprivation to T-cell exhaustion, ferroptotic vulnerability, and loss of cytotoxic function. CD36-mediated lipid uptake represents one important mechanism by which lipid-rich tumor microenvironments impair CD8+ T-cell function. Tumor-infiltrating CD8+ T cells can upregulate CD36 in response to lipid-rich conditions, leading to increased uptake of oxidized low-density lipoprotein (ox-LDL) and oxidized phospholipids that trigger lipid peroxidation and ferroptosis (65, 66). In preclinical tumor models, genetic deletion or pharmacological inhibition of CD36 restores cytotoxic T-cell function and can synergize with anti-PD-1 therapy (65). However, because much of this evidence is derived from non-GBM tumor models, CD36-dependent T-cell lipid toxicity should be presented as a biologically plausible immunometabolic mechanism that requires further GBM-specific validation. Cholesterol accumulation provides another route through which lipid metabolism contributes to T-cell exhaustion. Elevated intracellular cholesterol induces endoplasmic reticulum stress and activates X-box binding protein 1 (XBP1) signaling, which can promote the expression of inhibitory receptors such as PD-1 and 2B4 (64). Sustained activation of PD-1, TIM-3, LAG-3, and related inhibitory receptors further reinforces terminal exhaustion. PD-1 signaling also reshapes T-cell metabolism by suppressing glycolysis and promoting FAO through STAT3-dependent pathways (62, 67). Moderate FAO activation may transiently support T-cell persistence, whereas excessive dependence on FAO under lipid-rich and hypoxic conditions may deepen metabolic exhaustion. PPARα signaling is involved in this FAO program, and preclinical evidence suggests that modulation of PPAR signaling may improve anti-tumor T-cell responses and enhance immune checkpoint blockade in selected tumor contexts (68, 69). Whether this approach can be effectively translated to GBM remains to be determined. In addition to intrinsic lipid stress in T cells, other immunosuppressive cells within the TME aggravate T-cell exhaustion through lipid-mediated mechanisms. Myeloid-derived suppressor cells (MDSCs) exhibit enhanced fatty acid uptake and FAO, thereby exerting suppressive effects on CD8+ T cells (70). Lipid-loaded MDSCs inhibit T-cell proliferation and suppress IFN-γ and TNF-α production through ROS-dependent pathways (71). FATP2-mediated arachidonic acid uptake in MDSCs increases prostaglandin E2 (PGE2) synthesis and reinforces immunosuppressive signaling (72). PGE2 can directly suppress T-cell activation by reducing IL-2 production, decreasing CD28 expression, and weakening responsiveness to exogenous IL-2 (73). PGE2 signaling through EP2 and EP4 receptors may also increase PD-1 expression in infiltrating CD8+ T cells, providing a rationale for combining lipid mediator targeting with immune checkpoint blockade in appropriate tumor contexts (74, 75). Macrophages and regulatory T cells further contribute to lipid-mediated immune suppression. Tumor-associated macrophages can suppress T-cell activation through lipid metabolic remodeling, immunosuppressive cytokine production, and cholesterol handling programs. Inhibition of cholesterol efflux pathways in macrophages may promote M1-like polarization and enhance anti-tumor T-cell activity in experimental models (76). Regulatory T cells also depend on fatty acid synthesis and FAO to maintain their suppressive functions within nutrient-restricted TMEs (77). FASN-dependent lipid synthesis is required for Treg maturation and suppressive activity, suggesting that lipid metabolism supports both tumor cell survival and immune suppressor cell function (78). Collectively, lipid metabolic remodeling contributes to T-cell exhaustion through both intrinsic and extrinsic mechanisms. Intrinsically, lipid overload, cholesterol accumulation, CD36-mediated uptake, lipid peroxidation, ferroptotic vulnerability, and altered FAO impair CD8+ T-cell effector function. Extrinsically, lipid-reprogrammed MDSCs, macrophages, and Tregs reinforce an immunosuppressive niche through PGE2, ROS, cholesterol efflux, FAO, and lipid synthesis programs. These mechanisms support the concept that GBM immune suppression is partly maintained by a lipid-stressed TME. Targeting lipid uptake, cholesterol accumulation, FAO signaling, lipid peroxidation, or lipid-derived immunosuppressive mediators may provide rational immunometabolic strategies.

## 4 Lipid metabolism drives therapeutic resistance

**Figure 3:**
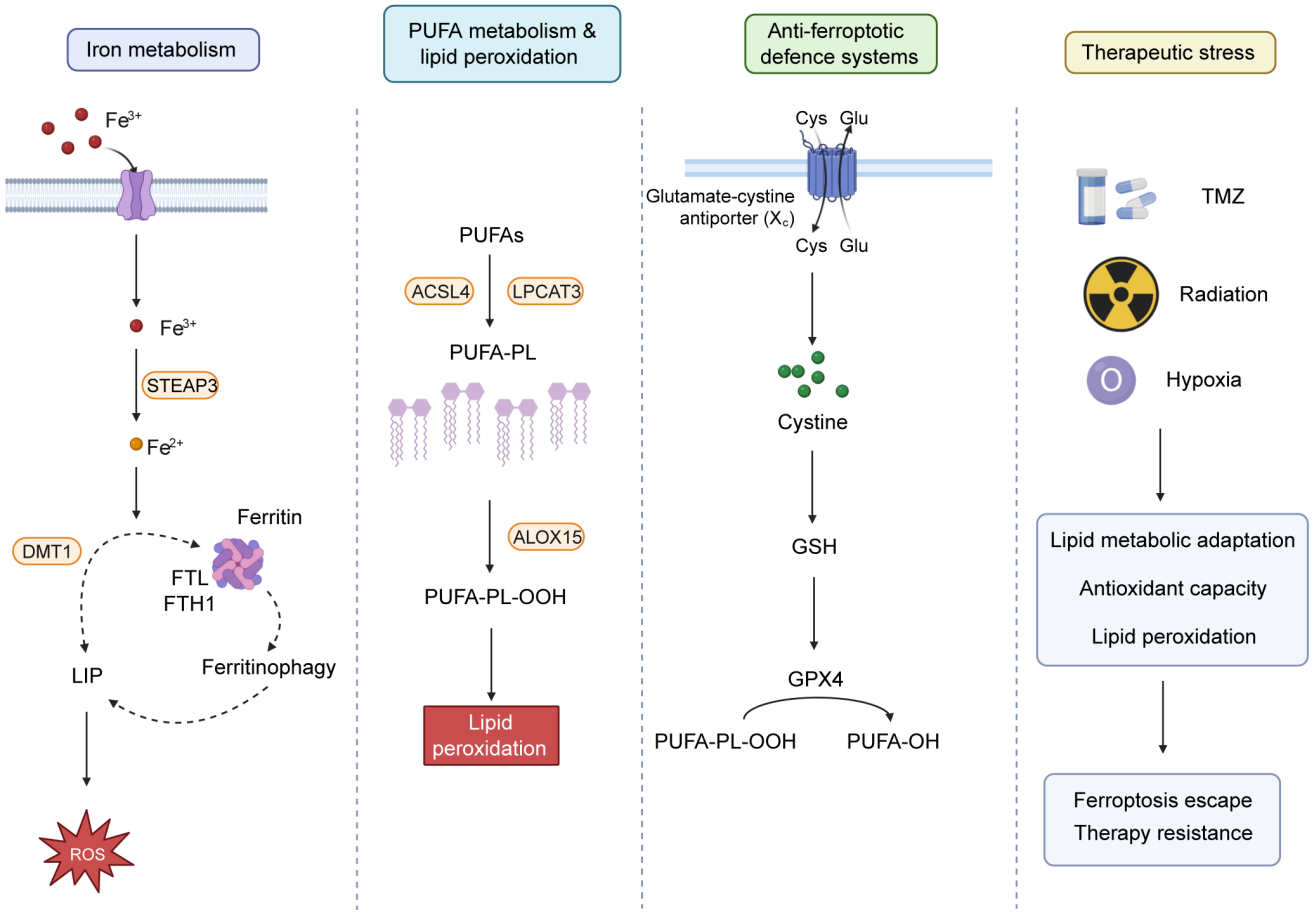
Lipid metabolism promotes ferroptosis resistance and therapy resistance in glioblastoma. This figure summarizes the role of lipid metabolism in regulating ferroptosis resistance and therapeutic resistance in GBM. Iron metabolism contributes to reactive oxygen species (ROS) generation through intracellular iron accumulation and ferritinophagy. Polyunsaturated fatty acids (PUFAs) are incorporated into membrane phospholipids by ACSL4 and LPCAT3 and subsequently oxidized by ALOX15, resulting in lipid peroxidation and ferroptotic signaling. GBM cells counteract ferroptosis through the glutamate-cystine antiporter (system xc^−^), glutathione (GSH) synthesis, and GPX4-mediated detoxification of lipid peroxides. Under therapeutic stress induced by temozolomide (TMZ), radiotherapy, and hypoxia, adaptive lipid metabolic reprogramming enhances antioxidant capacity and suppresses lipid peroxidation, thereby promoting ferroptosis escape and therapy resistance in GBM.

Aberrant lipid metabolic reprogramming is increasingly recognized as a major driver of therapeutic resistance in GBM. By reshaping ferroptosis sensitivity, redox homeostasis, membrane composition, and immune responses, lipid metabolism enables tumor cells to evade chemotherapy, radiotherapy, and immunotherapy induced cell death. Among these mechanisms, ferroptosis escape and the maintenance of lipid peroxidation balance have emerged as central adaptive strategies supporting tumor survival under therapeutic stress (**Figure 3**).

### 4.1 Ferroptosis escape and lipid peroxidation balance

Ferroptosis is an iron-dependent form of regulated cell death driven by overwhelming lipid peroxidation and oxidative membrane damage (79). The core molecular mechanism of ferroptosis involves the dynamic balance between oxidative damage and antioxidant defense systems (80). GBM cells exploit multiple lipid metabolic adaptations to maintain this balance and evade ferroptotic death under therapeutic stress (81). Intracellular iron accumulation and excessive lipid peroxide (LPO) generation are two essential prerequisites for ferroptosis induction. Iron promotes ferroptosis through Fenton chemistry and iron dependent lipid oxidation reactions (82). Ferrous iron (Fe2^+^) catalyzes the conversion of hydrogen peroxide into hydroxyl radicals, which initiate lipid radical chain reactions in PUFAs (83). PUFAs, particularly AA and adrenic acid (AdA), are highly susceptible to peroxidation and serve as major substrates for ferroptotic signaling (84). The incorporation of AA and AdA into membrane phospholipids is primarily mediated by ACSL4 and LPCAT3 (85, 86). ACSL4 catalyzes the activation of free AA and AdA into acyl-CoA derivatives, whereas LPCAT3 esterifies them into phosphatidylethanolamines within cellular membranes (87). These PUFA-containing phospholipids subsequently undergo oxidation by lipoxygenases such as ALOX15 to generate lethal lipid hydroperoxides (85, 86). Accumulation of lipid hydroperoxides ultimately disrupts membrane integrity and triggers ferroptotic cell death (86). To survive under oxidative stress, GBM cells develop multiple antioxidant systems to suppress lipid peroxide accumulation. Among these pathways, the system xc^-^/glutathione (GSH)/glutathione peroxidase 4 (GPX4) axis represents the central anti-ferroptotic defense mechanism (88). System xc^-^ imports extracellular cystine in exchange for intracellular glutamate, thereby supplying cysteine for GSH synthesis (89). GSH functions as an essential reducing cofactor required for GPX4 activity (90). GPX4 directly reduces phospholipid hydroperoxides into non-toxic lipid alcohols, thereby preventing the propagation of lipid peroxidation. Upregulation of GPX4 expression is a major mechanism by which tumor cells escape ferroptosis (91). Similarly, increased expression of SLC7A11 enhances cystine uptake and reinforces antioxidant capacity in tumor cells (89). Besides the GPX4 pathway, several GPX4 independent antioxidant systems also contribute to ferroptosis resistance. Ferroptosis suppressor protein 1 (FSP1) acts as an oxidoreductase that regenerates reduced coenzyme Q_10_ (CoQ_10_-H2) at the plasma membrane. Reduced CoQ_10_ functions as a lipophilic radical-trapping antioxidant that neutralizes lipid radicals and suppresses ferroptosis (92). The GCH1/BH4 pathway represents another endogenous antioxidant system capable of limiting membrane lipid peroxidation (93). BH4 efficiently traps peroxyl radicals and suppresses ferroptotic lipid damage (93). NRF2 is a master regulator of antioxidant signaling and a critical mediator of ferroptosis resistance in cancer cells (94). Activation of NRF2 transcriptionally upregulates multiple antioxidant and iron-handling genes, including SLC7A11, GPX4, FTH1, and ferroportin (95). Consequently, persistent NRF2 activation enables tumor cells to tolerate oxidative stress and resist ferroptosis-inducing therapies. Alterations in membrane lipid composition also critically determine ferroptosis sensitivity. Monounsaturated fatty acids (MUFAs) are substantially less susceptible to oxidation than PUFAs and therefore protect cells from ferroptosis (96). Stearoyl-CoA desaturase 1 promotes MUFA synthesis and enhances ferroptosis resistance by displacing oxidizable PUFAs from membrane phospholipids (81). ACSL3 similarly promotes incorporation of MUFAs into membrane phospholipids and protects tumor cells against ferroptosis (97, 98). In contrast, ACSL4 dependent enrichment of PUFA-containing phospholipids sensitizes cells to ferroptosis (85). Loss or suppression of ACSL4 is therefore a common mechanism underlying ferroptosis escape in resistant cancer cells. Tumor cells additionally reduce ferroptosis sensitivity through lipid storage and membrane remodeling mechanisms. Storage of PUFAs within LDs decreases their availability for membrane lipid peroxidation (99). Meanwhile, phospholipase-mediated removal of oxidized PUFA chains further limits propagation of lipid peroxidation (100). Hypoxia also contributes substantially to ferroptosis resistance in GBM. HIF-1α promotes fatty acid uptake and lipid storage, thereby reducing lipid peroxidation (101). In contrast, HIF-2α can increase PUFA production and sensitize specific tumor contexts to ferroptosis (102). These findings suggest that the balance between ferroptosis-promoting and ferroptosis suppressive lipid pathways dynamically determines therapeutic sensitivity in GBM. Under treatment pressure, GBM cells preferentially activate antioxidant defenses and MUFA enriched membrane remodeling programs to escape ferroptotic death. Targeting GPX4, FSP1, SLC7A11, NRF2, or MUFA metabolic pathways may therefore restore ferroptosis sensitivity and overcome therapeutic resistance in GBM.

### 4.2 Resistance to TMZ and radiotherapy

TMZ resistance and radioresistance remain major barriers to effective GBM treatment (103). Emerging evidence indicates that lipid metabolic reprogramming critically contributes to resistance against chemotherapy and radiotherapy in GBM (19). By remodeling membrane lipid composition, enhancing antioxidant defenses, and regulating ferroptosis sensitivity, GBM cells acquire adaptive mechanisms that support survival under therapeutic stress. One hallmark of TMZ-resistant GBM is profound alteration in fatty acid metabolism (104). TMZ-resistant GBM cells exhibit increased levels of long-chain fatty acids, including palmitate and stearate, together with reduced levels of PUFAs such as AA and docosahexaenoic acid (104). This lipid remodeling reduces membrane susceptibility to lipid peroxidation and ferroptotic damage. Upregulation of FASN is closely associated with enhanced fatty acid synthesis and TMZ resistance (104). FASN-mediated lipogenesis provides membrane components and metabolic substrates required for rapid tumor proliferation and stress adaptation (105). Inhibition of FASN sensitizes TMZ-resistant GBM cells to chemotherapy, highlighting its therapeutic relevance (106, 107). SREBPs also contribute to TMZ resistance by promoting *de novo* fatty acid synthesis (19). Activation of SREBP signaling enhances lipid biosynthesis and supports tumor cell survival during therapeutic stress (108). Increased expression of FATPs and FABP7 further promotes fatty acid uptake in resistant GBM cells (109). Enhanced lipid uptake provides metabolic flexibility and maintains energy production under treatment-induced stress conditions. Altered membrane lipid composition can additionally influence membrane fluidity, receptor localization, and drug transport. These membrane alterations may reduce intracellular TMZ accumulation and promote therapeutic resistance. Ferroptosis suppression represents another major mechanism underlying TMZ resistance (103). TMZ-resistant GBM cells frequently exhibit elevated expression of SLC7A11 and GPX4, thereby enhancing antioxidant defenses against lipid peroxidation (110). SLC7A11 mediated cystine uptake sustains glutathione synthesis and preserves redox homeostasis in resistant tumor cells. GPX4 detoxifies lipid hydroperoxides and protects membrane integrity, thereby preventing ferroptotic cell death (111). Inhibition of GPX4 markedly enhances TMZ cytotoxicity, particularly in MGMT positive GBM models (110). MicroRNAs regulating lipid metabolism also contribute to TMZ resistance. miR-670-3p suppresses ACSL4 expression, thereby reducing lipid peroxidation and decreasing ferroptosis sensitivity (112). Similarly, miR-18a inhibits ALOXE3-mediated lipid oxidation and promotes GBM cell survival (113). Long non-coding RNAs also modulate ferroptosis-related pathways associated with TMZ resistance. LINC01564 enhances NFE2L2 expression and promotes ferroptosis resistance in GBM cells (114). Steroid metabolism additionally contributes to TMZ resistance in GBM. TMZ-resistant GBM cells exhibit increased expression of steroidogenic enzymes including CYP11A1, CYP17A1, and CYP19A1 (115). Steroid metabolites such as DHEA and 17β-estradiol promote DNA repair and ROS clearance, thereby facilitating TMZ resistance (116). Activation of androgen receptor (AR) and NRF2 signaling by sex steroids further enhances tumor survival under chemotherapy (117). AA metabolism is another important contributor to therapeutic resistance. TMZ-resistant GBM cells exhibit increased expression of 5-LOX and elevated leukotriene production (118). PGE2 activates EP2/EP4 receptor signaling and promotes PI3K/Akt/mTOR pathway activation, thereby increasing TMZ tolerance (119). PGE2 additionally activates Wnt/β-catenin signaling and remodels the TME to support therapeutic resistance (120). Radiotherapy resistance is similarly associated with lipid metabolic adaptation and ferroptosis suppression. GBM cells develop strong antioxidant systems that neutralize radiation-induced oxidative stress and lipid peroxidation (121). Iron metabolism critically influences radiosensitivity through regulation of redox homeostasis and ferroptosis susceptibility. Transferrin receptor (TFRC), the major mediator of iron uptake, is strongly associated with radiation response in GBM (121). Conversely, ferroportin (FPN) mediated iron export decreases intracellular iron accumulation and reduces radiosensitivity (122). Iron oxide nanoparticles have been shown to increase intracellular labile iron pools and amplify radiation-induced lipid peroxidation (123). Transcriptomic analyses further reveal that radiation resistant GBM cells exhibit downregulation of multiple ferroptosis related genes (124). Collectively, these findings indicate that lipid metabolic remodeling and ferroptosis escape jointly promote resistance to TMZ and radiotherapy in GBM. Targeting lipid metabolism, antioxidant systems, and ferroptosis related pathways may therefore represent promising strategies to overcome therapeutic resistance in GBM.

## 5 Emerging technologies revealing lipid heterogeneity

The remarkable metabolic heterogeneity of GBM has increasingly driven the development of advanced technologies capable of resolving lipid metabolism at spatial, cellular, and molecular levels. Traditional bulk transcriptomic and metabolomic analyses often obscure critical regional and cellular differences within the TME (125). Recent advances in spatial metabolomics, mass spectrometry imaging (MSI), single-cell metabolomics, and multi-omics integration have enabled unprecedented characterization of glioma lipid heterogeneity. These technologies have substantially improved our understanding of how lipid metabolic programs differ across tumor regions, cellular states, and therapeutic conditions. Mass spectrometry-based metabolomics has become one of the most powerful tools for studying dysregulated lipid metabolism in GBM (126). Metabolomics aims to quantitatively analyze comprehensive sets of metabolites within biological systems and provide a snapshot of the metabolic state of tumors (127). In GBM research, metabolomics has enabled detailed characterization of lipid metabolic alterations associated with tumor progression, therapeutic resistance, and molecular subtypes. High-throughput analytical platforms including gas chromatography-mass spectrometry (GC-MS), liquid chromatography-mass spectrometry (LC-MS), and nuclear magnetic resonance spectroscopy are widely used for lipid profiling (128). These approaches enable quantitative detection of fatty acids, phospholipids, sphingolipids, sterols, and other bioactive lipid species across diverse biological samples (129). Targeted metabolomics focuses on predefined lipid metabolites and provides high sensitivity and quantitative precision (130). This strategy is particularly useful for validating specific lipid pathways associated with ferroptosis, FAO, or TMZ resistance. In contrast, untargeted metabolomics provides broader metabolite coverage and facilitates discovery of novel lipid signatures associated with GBM biology (131). Untargeted approaches have identified substantial alterations in phosphatidylcholines, phosphatidylethanolamines, sphingomyelins, and PUFAs in treatment resistant GBM (132). Importantly, metabolomic profiling has revealed profound lipidomic differences among distinct GBM molecular subtypes (133). Proneural GBMs exhibit enrichment of very-long-chain fatty acids and PUFA containing glycerophospholipids, whereas mesenchymal tumors display elevated glycerolipid accumulation (134). These subtype-specific lipid programs may contribute to differential therapeutic responses and ferroptosis susceptibility. Spatial metabolomics has emerged as a transformative technology for mapping lipid distributions within intact tumor tissues. Unlike bulk metabolomics, spatial metabolomics preserves tissue architecture and enables visualization of region-specific metabolic states. Mass spectrometry imaging (MSI) based platforms are currently the dominant technologies used for spatial metabolomics (125). MSI enables direct *in situ* detection of metabolites and lipids without requiring fluorescent labeling or antibody-based staining. Recent developments in matrix-assisted laser desorption ionization mass spectrometry imaging (MALDI-MSI) have substantially improved spatial resolution (135). Subcellular-resolution MALDI-MSI now allows investigators to characterize metabolic heterogeneity at the level of individual tumor cells and organelles (136). Spatial metabolomics has revealed marked regional differences in lipid accumulation, mitochondrial metabolism, and FAO within tumors (137). Hypoxic tumor regions frequently exhibit enhanced lipid droplet accumulation and altered phospholipid remodeling compared with normoxic regions. These observations support the concept that lipid metabolic adaptation is spatially organized within the GBM microenvironment. Single-cell metabolomics further extends spatial technologies by enabling direct measurement of metabolic states at cellular resolution (125). Single-cell approaches are especially valuable in GBM because tumor cells, immune cells, endothelial cells, and glioma stem cells display highly heterogeneous metabolic phenotypes. Single-cell metabolomics can distinguish lipid metabolic programs associated with stemness, proliferation, invasion, and therapy resistance. The SpaceM platform has enabled characterization of metabolic states in dissociated single cells using commercially available MALDI-MSI technology (138). However, cellular dissociation may itself alter endogenous metabolic states and remains an important technical limitation. Integration of metabolomics with transcriptomics has become increasingly important for interpreting lipid metabolic heterogeneity. Cross-omics integration enables direct association between metabolite distributions and transcriptional programs regulating lipid metabolism. Bioinformatic platforms such as METASPACE have greatly facilitated annotation and interpretation of spatial metabolomic datasets. METASPACE integrates spectral and spatial information to improve metabolite identification accuracy in MSI datasets (139). Such computational frameworks are particularly important because only a small proportion of imaging mass spectrometry signals can currently be confidently annotated (140). Pathway-enrichment tools including MetaboAnalyst, Reactome, and LION/web facilitate identification of dysregulated lipid metabolic pathways (141, 142). These platforms enable mapping of lipidomic alterations onto biological pathways including ferroptosis, FAO, phospholipid remodeling, and cholesterol metabolism. Network-based multi-omics analyses can further integrate metabolites, genes, proteins, and epigenetic regulators into unified functional networks (141). This systems-level approach is particularly valuable for understanding how oncogenic mutations reshape GBM lipid metabolism. Integration of genomics, epigenomics, transcriptomics, and metabolomics may ultimately facilitate construction of comprehensive metabolic atlases of GBM (133). Such atlases could identify region-specific metabolic vulnerabilities and guide precision metabolic therapies. Despite these advances, several technical limitations remain. Current single-cell metabolomics approaches face challenges related to sensitivity, metabolite coverage, ion suppression, and data processing complexity (143). Standardization of sample preparation, metabolite annotation, and cross-platform integration also remains insufficient (128). Nevertheless, rapidly evolving spatial and single-cell technologies are expected to fundamentally transform our understanding of GBM lipid biology. By resolving lipid metabolic heterogeneity across cellular states and microenvironmental niches, these technologies may uncover new biomarkers and therapeutic targets for precision GBM treatment.

## 6 Therapeutic opportunities and future perspectives

Targeting lipid metabolic reprogramming has emerged as a promising therapeutic strategy for GBM and other aggressive malignancies (144). Dysregulated lipid metabolism supports tumor proliferation, survival, therapeutic resistance, immune suppression, stemness maintenance, and stress adaptation within the tumor microenvironment (145). However, the current evidence remains uneven. Some strategies are supported mainly by GBM cell line, glioma stem-like cell, organoid, or xenograft data, whereas others are based on early clinical experience, drug repurposing evidence, or mechanistic findings from non-GBM tumor models. A central challenge is that GBM cells exhibit substantial metabolic plasticity. Inhibition of a single lipid pathway may induce compensatory activation of alternative programs, including exogenous lipid uptake, *de novo* lipogenesis, fatty acid oxidation (FAO), lipid droplet buffering, or cholesterol remodeling (146). Consequently, combination strategies integrating lipid metabolic targeting with TMZ, radiotherapy, ferroptosis induction, immune checkpoint blockade, or targeted therapy may be more suitable for overcoming metabolic adaptation and treatment resistance (145). Representative lipid metabolism directed interventions can be stratified according to the targeted metabolic module, treatment format, evidence stage, GBM specific evidence, and translational considerations (**Table 1**).

**Table 1:** Translational evidence for lipid metabolism-directed therapeutic strategies in GBM.

Lipid metabolic process	Molecular target or pathway	Representative agents or interventions	Monotherapy or combination strategy	Current evidence level	Evidence in GBM models or patients	Translational limitations and clinical considerations
De novo lipogenesis	FASN	Orlistat, cerulenin, C75, FASN blockade (147)	Single agent or combined with radiotherapy	Preclinical	GBM cell models and mouse models suggest reduced viability, apoptosis, autophagy, and enhanced radiosensitivity (10)	Selectivity, systemic metabolic toxicity, and brain delivery require further evaluation
Fatty acid oxidation	FAO, CPT1 related pathways	Etomoxir based FAO inhibition; FAO inhibition combined with AURKA inhibition	Combination therapy	Preclinical	Combination of AURKA inhibition and FAO inhibition has been reported to suppress GBM growth in preclinical models (148)	Optimal therapeutic window and metabolic compensation remain unclear
Lipid uptake and trafficking	CD36, FATPs, FABPs, FABP7	CD36 inhibition, FABP inhibitors, FATP2 inhibition	Single agent or immunotherapy combination	Mostly preclinical; some cross cancer rationale	GBM evidence supports roles of lipid transporters and FABP7 in lipid uptake, migration, hypoxic adaptation, and slow cycling cells (17)	Some immune related evidence comes from non GBM models and should be clearly labeled
Cholesterol metabolism and membrane remodeling	HMGCR, LDLR, LXR, lipid rafts	Statins, LXR agonists, cholesterol modulation	Single agent or combined with radiotherapy, chemotherapy, or immunotherapy	Preclinical and early clinical or repurposing evidence	Statins and LXR related approaches show anti GBM activity in models (149); Clinical benefit remains inconsistent	Existing clinical data are not definitive; BBB penetration, dose, tumor subtype, and combination context are important
Cholesterol esterification and lipid droplet formation	SOAT1 or ACAT1	Avasimibe, SOAT1 inhibition	Single agent or combined with radiotherapy or ferroptosis induction	Preclinical	SOAT1 inhibition suppresses GBM growth and may increase ferroptosis sensitivity (150)	Cell type specificity, macrophage expression, and normal brain lipid homeostasis need careful assessment
Lipid droplet biogenesis and utilization	DGAT1, lipophagy, LD turnover	DGAT1 inhibitors, LD formation blockade	Single agent or combination therapy	Preclinical	DGAT1 inhibition disrupts lipid homeostasis, increases oxidative stress, and may impair radioresistant or stress adapted GBM cells (151)	LD heterogeneity may influence response; Biomarkers are needed
Ferroptosis regulation	SLC7A11, GPX4, ACSL4, lipid peroxidation balance	Ferroptosis inducers, GPX4 or system xc inhibition, radiotherapy or immunotherapy combinations	Combination therapy	Preclinical	GBM models support lipid peroxidation and ferroptosis as treatment relevant vulnerabilities (152)	Need to avoid neurotoxicity and identify tumors with ferroptosis permissive lipid states
Lipid mediated immunosuppression	CD36, PGE2, FAO in immune cells, cholesterol efflux	CD36 blockade, COX2 or PGE2 axis inhibition, FAO modulation, checkpoint blockade combinations (153)	Combination therapy	Mostly preclinical; cross cancer evidence important	GBM TME contains lipid remodeled macrophages and exhausted T cells, but several intervention studies are extrapolated from other cancers	Must distinguish tumor cell targeting from immune cell reprogramming; GBM specific validation is needed
Drug delivery and repurposing	BBB penetration and lipid based delivery systems	Nanostructured carriers, lipid nanoparticles, repurposed lipid metabolic drugs	Combination or delivery enhancing strategy	Preclinical and early translational	Nanocarriers may improve CNS delivery and drug stability (154)	Clinical feasibility depends on safety, reproducibility, and integration with standard radiochemotherapy

This table summarizes representative therapeutic strategies targeting major lipid metabolic processes involved in GBM progression, stress adaptation, immune suppression, and therapy resistance. The listed interventions are organized according to lipid metabolic process, molecular target or pathway, representative agents, treatment format, current evidence level, GBM-specific evidence, and translational considerations. Evidence levels are broadly classified as preclinical, early translational, early clinical, repurposing-based, or cross-cancer rationale. When supporting data are derived mainly from non-GBM tumor models, the table indicates that these findings should be interpreted as mechanistic or translational rationale rather than direct GBM therapeutic evidence.

One therapeutic direction involves targeting lipid uptake in tumor cells and immunosuppressive cells within the tumor microenvironment (155). CD36, a major fatty acid transporter, contributes to fatty acid uptake, lipid droplet accumulation, metastatic progression, immune dysfunction, and therapy resistance in multiple tumor contexts (156). Preclinical non-GBM tumor models suggest that CD36 blockade can reduce tumor progression, enhance chemotherapy sensitivity, and improve anti-tumor immunity (157–160). Although direct GBM evidence remains limited, findings from non-GBM tumor models suggest that CD36-mediated lipid uptake can be therapeutically targeted to reduce tumor progression and improve anti-tumor immunity. Given the expression of CD36 in GBM-associated tumor and immune compartments, this pathway provides a biologically plausible but still incompletely validated therapeutic direction for GBM. FABPs and FATPs have similarly emerged as candidate regulators of lipid transport and immune metabolism (161). FABP7 has relatively direct relevance to GBM because it regulates fatty acid uptake, lipid droplet formation, hypoxic adaptation, and migratory behavior in GBM models. By contrast, several therapeutic observations involving broader FABP inhibition or FATP2 blockade are derived from non-GBM tumor or immunosuppressive myeloid cell models (162, 163). These studies provide cross-cancer mechanistic rationale for targeting lipid trafficking, but their therapeutic relevance in GBM should be validated in brain tumor models that preserve tumor immune interactions. Targeting DNL is another promising therapeutic direction in GBM. Key lipogenic enzymes including ACLY, ACC, and FASN are frequently upregulated in aggressive tumors and contribute to tumor growth and therapeutic resistance (164). Inhibition of ACLY suppresses acetyl-CoA production and sensitizes resistant tumors to targeted therapy (164). Similarly, ACC inhibitors such as TOFA and ND-646 have shown strong anti-tumor effects in combination with chemotherapy and targeted agents in preclinical models (165). However, for several ACLY or ACC inhibitors, direct GBM evidence remains limited and should be distinguished from cross-cancer data. FASN has attracted particular interest because it functions as a central regulator of fatty acid synthesis and membrane lipid remodeling. Pharmacological FASN inhibitors, including Orlistat and TVB series compounds, have demonstrated potent anti-tumor effects in multiple cancer models (166). Importantly, combining FASN inhibition with chemotherapy, targeted therapy, or radiotherapy markedly enhances therapeutic efficacy (167). Because GBM cells strongly depend on membrane remodeling and lipid biosynthesis under stress conditions, FASN-targeted therapy may be particularly relevant in recurrent and treatment-resistant disease. Targeting cholesterol metabolism also represents a promising therapeutic opportunity (168). Statins, which inhibit HMG-CoA reductase and suppress cholesterol biosynthesis, have demonstrated anti-proliferative and pro-apoptotic effects in GBM models (169). Cholesterol depletion additionally alters membrane fluidity and disrupts receptor mediated oncogenic signaling. Because cholesterol-rich lipid rafts regulate EGFR, PI3K/Akt, and immune checkpoint signaling, disrupting cholesterol homeostasis may simultaneously inhibit tumor growth and immune evasion (170). Nevertheless, existing clinical or observational evidence for statins and other systemic lipid-modifying agents in GBM remains insufficient to support definitive therapeutic conclusions. Future studies should clarify drug exposure in the central nervous system, dose requirements, patient selection, and whether cholesterol-modulating agents are more effective in combination with radiotherapy, TMZ, or immunotherapy. Ferroptosis induction has emerged as another highly promising therapeutic strategy for GBM (171). IFN-γ released by immunotherapy-activated CD8+ T cells downregulates SLC3A2 and SLC7A11 expression in tumor cells, thereby enhancing ferroptosis-specific lipid peroxidation (172). Cystinase, an engineered enzyme that induces oxidative stress and promotes ferroptosis, synergizes with anti-PD-L1 therapy to enhance antitumor immunity in preclinical tumor models (172). These findings support a mechanistic link between anti-tumor immunity and ferroptosis, but their direct therapeutic applicability to GBM requires further validation. Future GBM studies should identify ferroptosis-permissive lipid states, define biomarkers of lipid peroxidation sensitivity, and evaluate neurotoxicity risks when combining ferroptosis induction with radiotherapy or immunotherapy. Lipid metabolism also intersects with immunotherapy responsiveness. PCSK9 inhibitors, which regulate cholesterol metabolism, have shown synergy with anti-PD-1 therapy in non-GBM tumor models and have entered early clinical evaluation in selected cancer contexts (173). Fatty acid metabolic pathways have also emerged as highly druggable therapeutic targets. Inhibiting DNL via ACLY inhibitors such as EVT0185 and bempedoic acid sensitizes hepatocellular carcinoma to anti-PD-L1 therapy (174). These findings should be interpreted as cross-cancer translational rationale rather than direct GBM evidence. For GBM, it remains necessary to test whether targeting PCSK9, ACLY, FATP2, CD36, or lipid-derived mediators can overcome the immune-suppressive barriers created by glioma-associated myeloid cells, exhausted T cells, regulatory T cells, and the blood-brain barrier. Although current clinical outcomes remain limited, these studies support the feasibility of targeting lipid metabolism in GBM therapy. Future therapeutic strategies will likely require rational combination approaches integrating metabolic targeting with immunotherapy, radiotherapy, and ferroptosis induction. Advances in spatial transcriptomics, single-cell metabolomics, and multi-omics integration are expected to facilitate patient stratification and precision metabolic therapy. Because GBM exhibits profound metabolic heterogeneity and plasticity, identifying context specific metabolic vulnerabilities will be critical for future clinical translation. Overall, targeting lipid metabolism represents a promising avenue for overcoming therapeutic resistance and improving clinical outcomes in GBM.

## 7 Conclusion

Collectively, accumulating evidence indicates that lipid metabolic reprogramming is not merely a secondary consequence of malignant transformation, but rather a central driver of GBM progression, immune suppression, and therapeutic resistance (175). Unlike normal brain tissue, GBM cells exhibit remarkable lipid metabolic plasticity, allowing dynamic adaptation to hypoxia, nutrient deprivation, oxidative stress, and therapeutic exposure. This adaptive metabolic network is supported through coordinated enhancement of DNL, exogenous lipid uptake, cholesterol remodeling, FAO, and lipid droplet formation (175). Importantly, lipid metabolic remodeling extends beyond tumor intrinsic metabolism and profoundly shapes the immunosuppressive TME (155). Accumulating evidence further indicates that lipid remodeling not only sustains membrane biosynthesis and energy production, but also regulates ferroptosis sensitivity, stemness maintenance, and immunosuppressive signaling. Recent advances in spatial transcriptomics, single-cell metabolomics, and multi-omics integration have substantially improved our understanding of lipid heterogeneity within GBM. Importantly, targeting lipid metabolic pathways, including fatty acid uptake, DNL, cholesterol metabolism, lipid droplet formation, and ferroptosis regulatory networks, has demonstrated promising therapeutic potential in preclinical GBM models. Nevertheless, profound metabolic heterogeneity and adaptive plasticity remain major challenges limiting the clinical translation of metabolism-targeted therapies. Future therapeutic strategies will likely require rational combination approaches integrating metabolic targeting with immunotherapy, radiotherapy, ferroptosis induction, and multi-omics guided patient stratification. Collectively, continued investigation into lipid metabolic plasticity and TME interactions may provide new opportunities for overcoming therapeutic resistance and improving outcomes for patients with GBM.
